# Retrospective Study of 32 Kissing Aneurysms: Radiological Detection, Microsurgical Clipping, and Functional Outcomes

**DOI:** 10.2174/0115734056411946251202071718

**Published:** 2026-02-02

**Authors:** Alejandro Serrano-Rubio, Sharon-Paola Garcia-Trujillo, Brenda-Susana Hernandez-Barrera, Ana-Margarita Martinez-Caceres, Ambar-Elizabeth Riley-Moguel, Rafael Sanchez-Mata, Daniel Figueroa Zelaya, Julian-Moises Enriquez-Alvarez, Ernesto Roldan-Valadez, Edgar Nathal

**Affiliations:** 1 Department of Vascular Neurosurgery, National Institute of Neurology and Neurosurgery “Manuel Velasco Suárez”, 14267, Mexico City, Mexico; 2 Division of Research, Instituto Nacional de Rehabilitación “Luis Guillermo Ibarra Ibarra”, 14389, Mexico City, Mexico; 3 I.M. Sechenov First Moscow State Medical University (Sechenov University), Radiology Department, 119992, Moscow, Russia

**Keywords:** Kissing aneurysms, Intracranial aneurysms, Microsurgical clipping, Subarachnoid hemorrhage, Internal carotid artery, Digital subtraction angiography, Cerebrovascular surgery, Vascular imaging

## Abstract

**Introduction:**

Kissing aneurysms (KA) are a rare variant of multiple intracranial aneurysms characterized by two adjacent aneurysms arising from separate vascular origins with adherent domes. Their atypical morphology often leads to misinterpretation as a single bilobulated aneurysm, which complicates diagnosis and surgical planning. Preoperative distinction between the aneurysms is especially crucial when rupture occurs. This study aimed to analyze the clinical, radiological, and surgical characteristics of 32 patients with KA treated microsurgically over two decades and provide a narrative review of the literature to contextualize our findings.

**Methods:**

A retrospective observational study was conducted on patients diagnosed with KA and treated by microsurgical clipping between January 2004 and October 2024. Imaging modalities included computed tomography (CT), CT angiography, and digital subtraction angiography. Microsurgical approaches were tailored to aneurysm location and rupture status. Functional outcomes were assessed using the modified Rankin scale (mRS).

**Results:**

KA accounted for 2.3% of all aneurysms treated during the study period. The most frequent site was the posterior communicating artery-anterior choroidal artery segment (59.37%). Preoperative identification of KA was achieved in 87.5% of cases. Intraoperative rupture occurred in 25%. Favorable outcomes (mRS 0–2) were observed in 87.5% of patients. Surgical approach selection, vascular control, and intraoperative Doppler guidance were critical to success.

**Discussion:**

KA remains a diagnostic challenge, with a portion of cases confirmed only intraoperatively. Microsurgical clipping of these complex lesions requires precise dissection, temporary vascular control, and intraoperative vascular imaging to ensure complete occlusion and vessel preservation. Age appears to be a risk factor for rupture, underscoring the need for vigilant monitoring in older patients.

**Conclusion:**

Kissing aneurysms remain a diagnostic and surgical challenge due to their complex anatomy and high rupture risk. Accurate imaging, individualized surgical planning, and intraoperative vessel assessment are essential to optimize outcomes.

## 1 INTRODUCTION

Kissing aneurysms (KA) are a rare and distinct subset of multiple intracranial aneurysms. They are defined as two closely adjacent aneurysms arising from separate arterial origins with partially adherent walls. This unique configuration can lead to diagnostic confusion, as these lesions are often misinterpreted as single bilobed aneurysms on conventional imaging [1, 2]. First described by Jefferson in 1978, KA account for less than 1% of all aneurysms, though reported incidence ranges from 0.1% to 3% depending on the imaging modality and surgical experience [3, 4].

The most frequently affected site is the internal carotid artery (ICA), particularly at the junction with the posterior communicating artery (PComA) and anterior choroidal artery (AChA). Other reported locations include bilateral anterior cerebral arteries (ACA), the anterior communicating artery (AComA), middle cerebral artery bifurcations, and proximal segments of the basilar artery with fenestration [5-10].

Preoperative identification is often challenging, especially when one aneurysm ruptures and the other remains unrecognized. This is clinically significant, as 57% of KA cases are not diagnosed correctly before surgery, potentially leading to suboptimal treatment strategies [11, 12]. Digital subtraction angiography (DSA) remains the gold standard for detection, particularly in evaluating dome projections and neck relationships. However, even with advanced imaging, determining the ruptured aneurysm can be difficult when multiple lesions are present, especially in the context of subarachnoid hemorrhage [13].

In the present study, we report our institutional experience managing 32 patients with KA over two decades, all of whom were treated with microsurgical clipping. We also review the relevant literature to provide an integrated perspective on the diagnostic pitfalls, surgical techniques, and outcomes associated with this rare but clinically significant vascular entity.

## 2 MATERIALS AND METHODS

### 2.1 Study Design and Setting

This retrospective observational study was conducted at the National Institute of Neurology and Neurosurgery “Manuel Velasco Suárez” in Mexico city. It included all patients diagnosed with kissing aneurysms (KA) who underwent microsurgical clipping between January 2004 and October 2024. The study was approved by the institutional review board (IRB authorization number 187/24) and conducted in accordance with institutional ethical guidelines and the principles outlined in the Declaration of Helsinki.

### 2.2 Patient Selection and Eligibility Criteria

Patients were eligible if they met the following criteria:

(1) Presence of two adjacent aneurysms arising from separate vascular origins with partially adherent domes, as confirmed by DSA and/or CT-A or intraoperative findings.

(2) Surgical management *via* open microsurgical clipping.

(3) Availability of preoperative imaging and complete follow-up data.

### 2.3 Exclusion Criteria

The exclusion criteria were as follows:

(1) Patients with endovascular-only treatment

(2) Unclear documentation of aneurysmal morphology

(3) Patients without preoperative DSA or CT-A

### 2.4 Imaging Protocol and Diagnosis

**Fig. (1):**
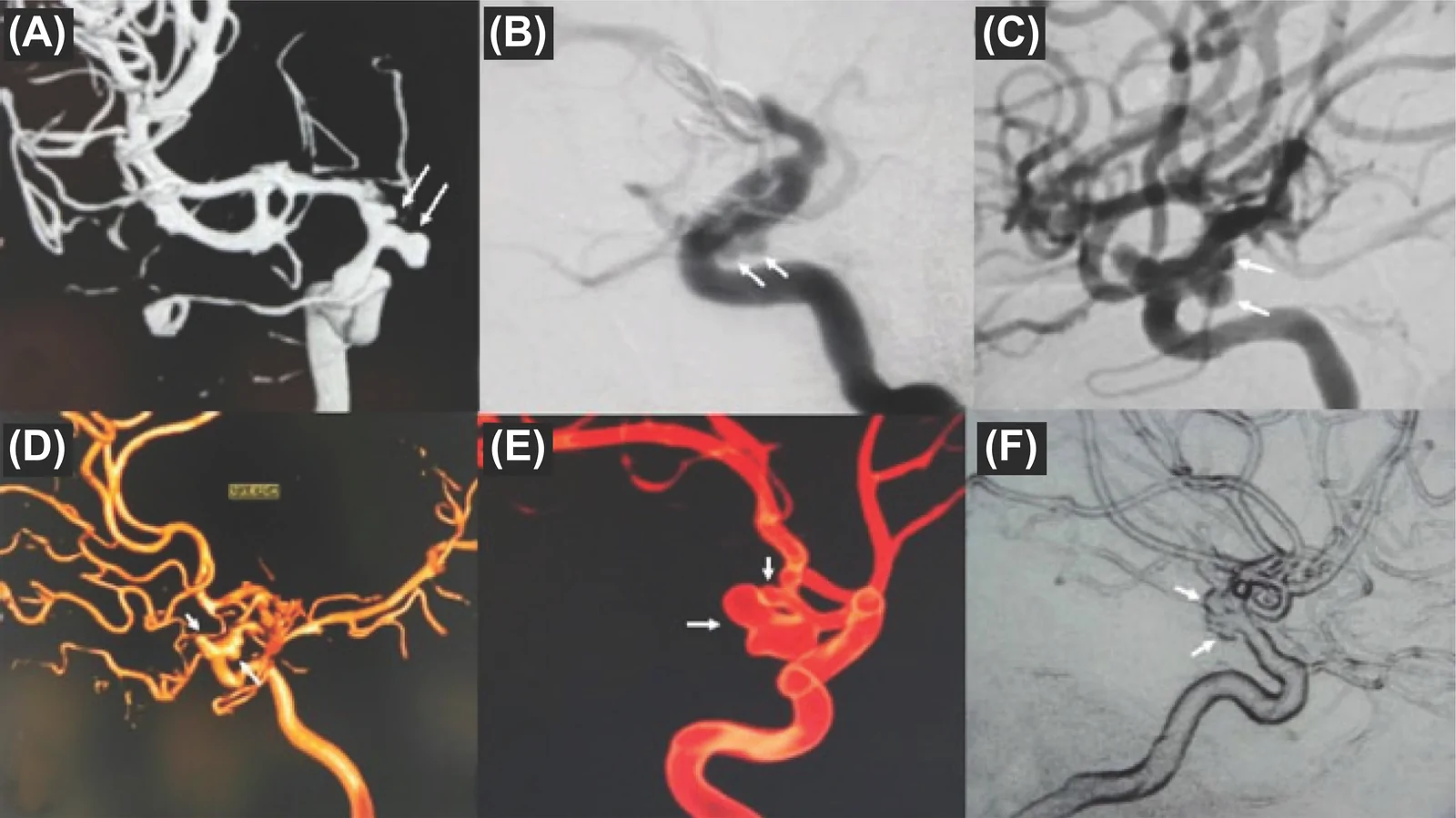
(**A**) Typical localization of the most common anatomical site for kissing aneurysms in the posterior communicating artery–anterior choroidal artery (PComA–AChA) segment on a 3D CT angiography reconstruction. (**B**) The radiological appearance on digital subtraction angiography (DSA) in a lateral projection of a case of kissing aneurysms in the paraclinoid region. (**C**) Digital subtraction angiography (DSA) illustrating another case of kissing aneurysm with different dome projections; arrows indicate the anatomical position of the aneurysmal domes and their spatial relationship. (**D**) Computed tomography angiography (CTA) reconstruction, highlighting the potential for misclassification as bilobed lesions. (**E**) In some cases, the diagnosis of this rare entity can be made with CTA reconstructions. (**F**) Three-dimensional DSA reconstructions were useful in delineating dome projection and neck morphology; arrows indicate the anatomical position of the aneurysmal domes.

All patients underwent cranial computed tomography (CT) as an initial evaluation tool. In non-emergency settings, computed tomography angiography (CT-A) and digital subtraction angiography (DSA) were used for diagnostic confirmation and surgical planning. DSA was particularly critical for visualizing aneurysm morphology, dome orientation, neck configuration, and vascular relationships (Fig. **1**). In cases presenting with subarachnoid hemorrhage (SAH), DSA also aided in identifying the ruptured aneurysm.

### 2.5 Surgical Approaches

All surgeries were performed by senior neurosurgeons using standard microsurgical techniques. Approach selection was individualized based on aneurysm location, rupture status, and cerebral edema:

- **The pterional approach:** It was used in patients with ruptured aneurysms and tomographic evidence of cerebral edema.
- **Minipterional approach:** It was selected for unruptured aneurysms or ruptured lesions in patients with preserved neurological status and no edema [14].
- **Mini-anterior interhemispheric approach (MAIA):** It was employed exclusively for kissing aneurysms located at the distal anterior cerebral artery [15].

Intraoperative techniques included the following:

- Dissection of aneurysm domes and necks with preservation of parent arteries.
- Use of pilot clips to control bleeding points.
- Temporary proximal and distal vascular control applying a temporary clip in the proximal or distal parent artery when needed.
- Microvascular Doppler and fluorescein video-angiography to assess arterial patency before and after clip placement.
- Postoperative CT-A in all cases to confirm complete occlusion.

### 2.6 Variables and Outcome Measures

The following data were collected: patient demographics, aneurysm location, rupture status, imaging findings, surgical approach, intraoperative rupture, vascular anomalies, and postoperative outcomes. Functional outcome was assessed using the modified Rankin scale (mRS) at discharge.

### 2.7 Statistical Analysis

Descriptive statistics were used to summarize demographic, surgical, and clinical outcome variables. Categorical variables (*e.g*., sex, rupture status, location) were compared using Fisher’s exact test due to small sample sizes. Differences in age between ruptured and unruptured groups were assessed using the Mann-Whitney U test, a non-parametric alternative suitable for small sample distributions. A two-sided *p*-value of <0.05 was considered statistically significant. All analyses were performed using Python (SciPy statistical package, version 1.11).

## 3 RESULTS

### 3.1 Patient Demographics and Clinical Characteristics

A total of 32 patients with kissing aneurysms (KAs) were included in this retrospective case series. The cohort was predominantly female (n = 27, 84.37%) with a smaller male subset (n = 5, 15.62%). The mean age was 55.87 years (range: 21–73). Table **1** summarizes the clinical, anatomical, and surgical characteristics of the series.

At initial presentation, 10 patients (35.7%) had ruptured aneurysms, while 18 patients (64.3%) had unruptured lesions. Among ruptured cases, the Fisher scale distribution was as follows: grade I (20%), grade II (60%), grade III (10%), and grade IV (10%). The Hunt and Hess (H&H) scores on admission were as follows: grade I (n = 4), grade II (n = 5), and grade IV (n = 1).

### 3.2 Aneurysm Location and Imaging Accuracy

The most frequent anatomical site was the posterior communicating artery-anterior choroidal artery (PComA–AChA) segment (Fig. **1A**), involved in 17 cases (60.7%). Other locations included distal ACA (n = 2), AComA (n = 1), and paraclinoid aneurysms in dorsal, ventral, or tandem configurations (n = 8) (Fig. **1B**).

In 84% of cases (n = 24), the diagnosis of KA was established preoperatively using DSA (Fig. **1C**) and CT-A (Fig. **1D**), aided by 3D reconstructions (Fig. **1E**). In four patients (14.3%), the kissing configuration was confirmed intraoperatively. Three-dimensional DSA reconstructions were useful in delineating dome projection and neck morphology (Fig. **1F**).

Statistical analysis showed no significant association between aneurysm location (PcomA-AChA *vs*. other sites) and rupture risk (*p* = 1.00; Fisher’s exact test).

### 3.3 Surgical Approach and Intraoperative Findings

Microsurgical clipping was performed in all 32 patients. The minipterional approach was used in 13 cases (46.4%), followed by the pterional approach in 12 cases (42.8%). The mini-anterior interhemispheric approach (MAIA) was employed in two cases (7.1%), and a single patient (3.5%) required a larger Dandy-type craniotomy due to severe cerebral edema.

Intraoperative rupture occurred in 8 patients (28.5%) and was managed using proximal vascular control, applying a temporary clip in the proximal parent artery and clip adjustment. Vascular anomalies, such as fenestrations, were identified in two patients (7.1%). Intraoperative patency of parent and perforating arteries was verified with microvascular Doppler and fluorescein videoangiography. The size of the aneurysm was not associated with the functional outcome in this series.

### 3.4 Functional Outcomes

The modified Rankin scale scores before surgery were as follows:

- mRS 0 in 15 patients (46.88%)
- mRS 1 in 10 patients (31.25%)
- mRS 2 in 3 patients (9.38%)
- mRS 3 in 2 patients (6.25%)
- mRS 4 in 0 patients (0%)
- mRS 5 in 2 patients (6.25%)

Most patients experienced a favorable recovery after treatment, elucidated as follows:

- mRS 0 in 14 patients (43.75%)
- mRS 1 in 12 patients (37.5%)
- mRS 2 in 2 patients (6.25%)
- mRS 3 in 2 patients (6.25%)
- mRS 4 in 1 patient (3.13%)
- mRS 5 in 0 patients (0%)
- mRS 6 in 1 patient (3.13%)

Four patients (12.5%) had poor outcomes (mRS 3, 4, or 6). Among them, one patient presented in poor neurological condition (H&H IV) and died from medical complications; another developed hemiplegia after an unnoticed AChA occlusion, and clip repositioning was performed, but a small infarction occurred. One patient developed vasospasm and internal capsule infarction on day 7 post-SAH. CT angiography, along with Doppler ultrasound and cerebral angiography, was performed to determine the degree of vasospasm. The fourth patient developed vasospasm, which was confirmed with cerebral angiography, and subsequently, hydrocephalus, requiring a ventricular shunt valve.

No significant association was found between sex and rupture status (*p* = 1.00, Fisher’s exact test). However, age differed significantly between ruptured and unruptured groups; the median age in ruptured patients was 55 years, compared to 54 years in unruptured cases (*p* = 0.0176, Mann–Whitney U test), suggesting that age may play a role in aneurysm instability. In one case, the misdiagnosis resulted in the selection of a suboptimal surgical approach and failure to anticipate dual aneurysm anatomy, leading to technical complications in the dissection and clipping of the distal aneurysm. However, the poor outcomes were more related to the patients' poor neurological status and initial Fisher score upon admission than to the technical complications presented.

### 3.5 Representative Cases

**Fig. (2):**
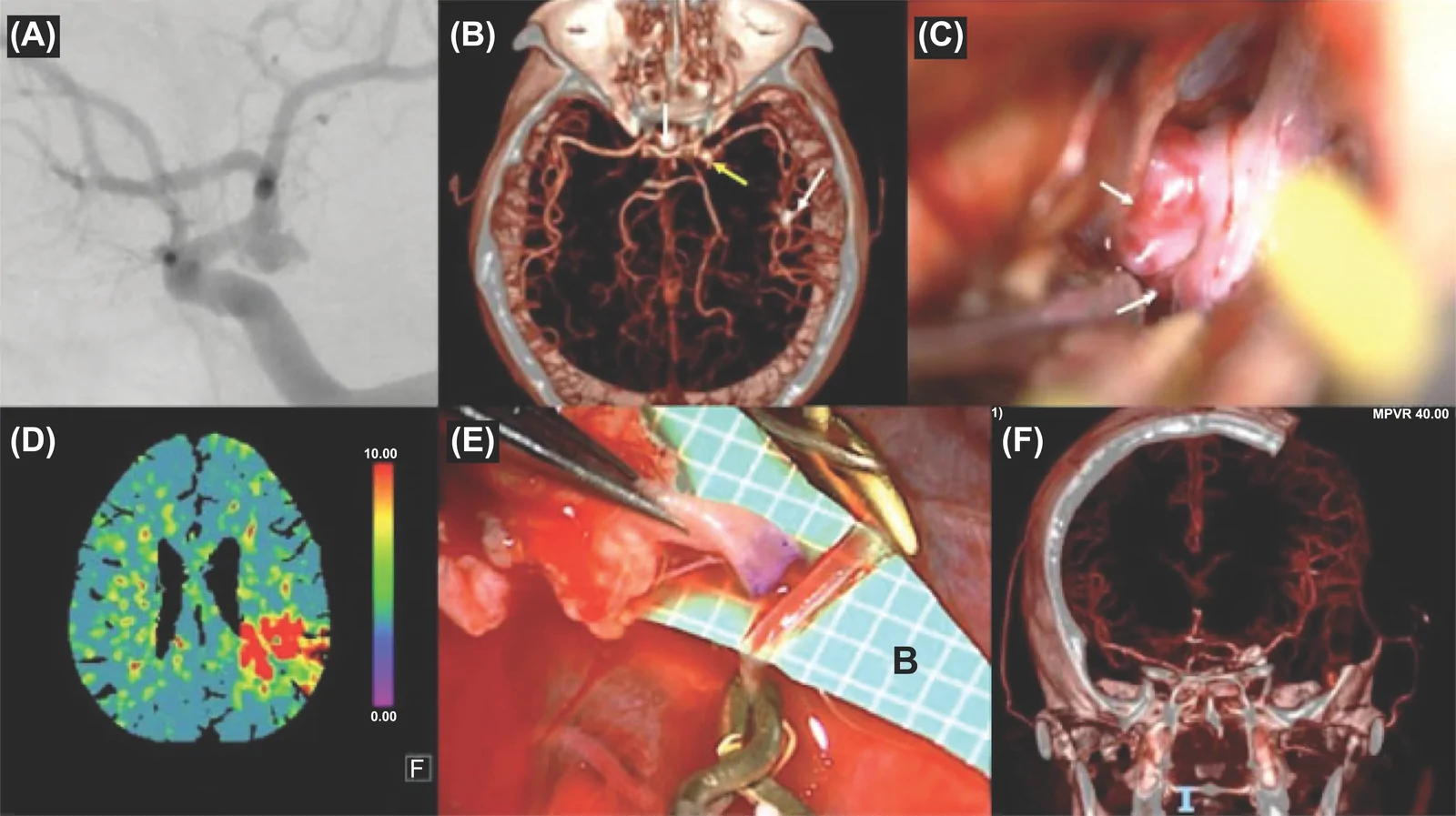
Case 1. **(A)** Oblique-view DSA reveals a bi-lobed configuration consistent with kissing aneurysms. **(B)** Preoperative CTA demonstrates migrated embolization coils (white arrows) and a persistent aneurysm sac (yellow arrow). **(C)** Intraoperative view confirms the presence of two separate aneurysms (arrows). **(D)** Perfusion CT shows prolonged transit time in the frontoparietal region related to the coil migration. **(E)** A low-flow superficial temporal artery–middle cerebral artery (STA–MCA) bypass was performed to support perfusion. **(F)** Postoperative CTA reveals restored MCA circulation; the decompressive craniectomy is evident.

Case 1 (Fig. **2**): A 42-year-old male presented with seizures and was diagnosed with Fisher II SAH. Imaging revealed on DSA (Fig. **2A**) kissing aneurysms at the PComA and AChA segments as observed in the 3D CTA reconstruction (Fig. **2B**). Coil embolization failed due to coil migration. Microsurgical clipping was performed (Fig. **2C**). Perfusion CT showed prolonged transit time in the left frontoparietal region (Fig. **2D**). The surgical procedure included removal of migrated coils and STA–MCA bypass (Fig. **2E**). In the control study, it was noted that MCA circulation was restored (Fig. **2F**). The patient was discharged neurologically intact on postoperative day 7.

**Fig. (3):**
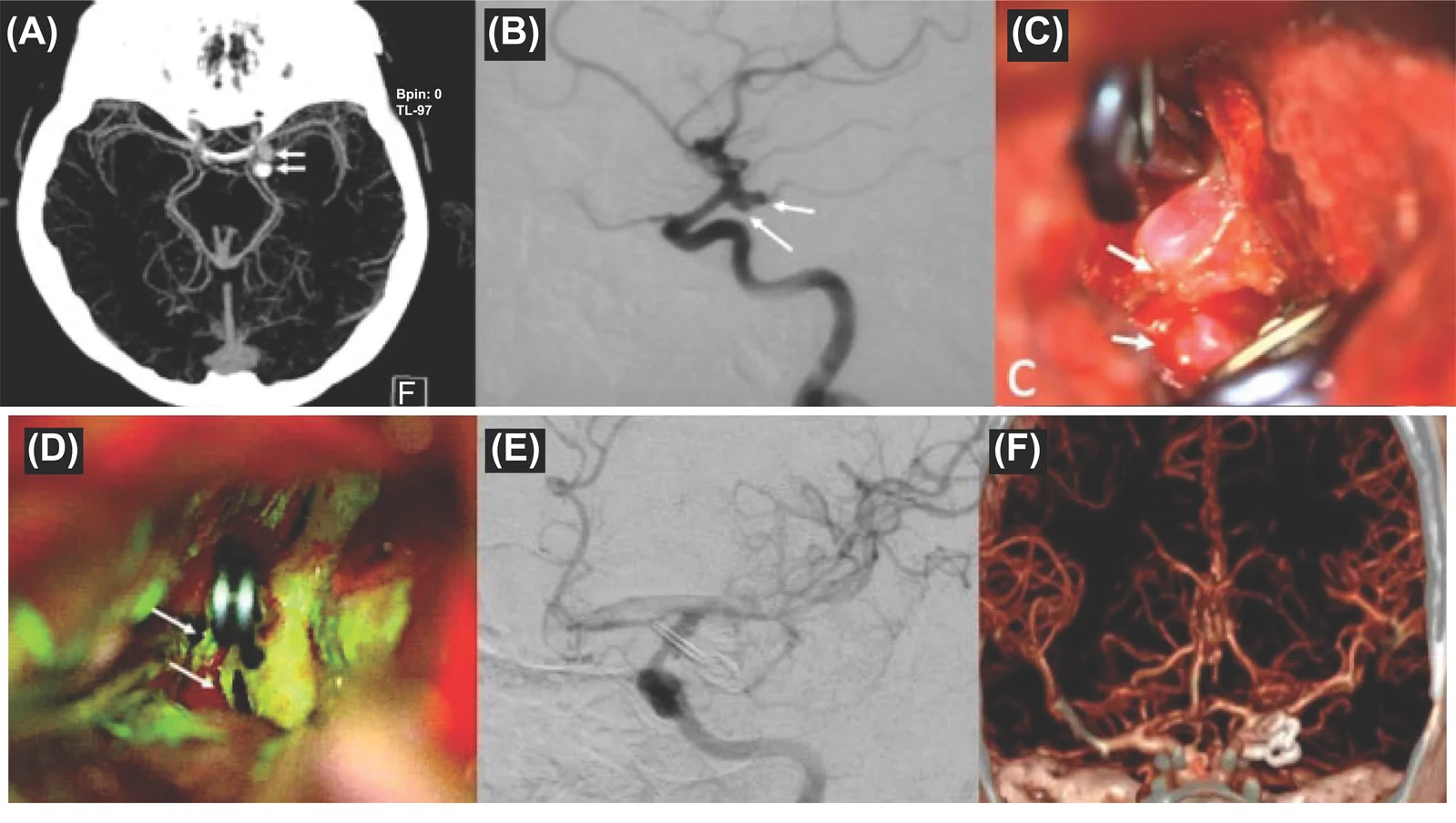
Case 2. (**A**) Axial CTA reconstruction showed a bilobulated aneurysmatic lesion on the left internal carotid artery (ICA). (**B**) DSA images confirm kissing aneurysms located at the PComA and AChA segments of the left internal carotid artery (ICA). (**C**) Intraoperative photograph displays both aneurysms dissected under temporary ICA clipping. (**D**) Fluorescein videoangiography confirms patency of the PComA and AChA (arrows). (**E**) Postoperative DSA shows complete occlusion of the aneurysms with patency of the parental vessel. (**F**) CTA confirms complete clipping of both aneurysms and adequate position of the clips.

Case 2 (Fig. **3**): A 64-year-old hypertensive female presented with headache and visual disturbances. Initially, a CTA showed a bilobulated aneurysmatic lesion (Fig. **3A**), and in DSA, it was confirmed as KA at the ICA–PComA–AChA (Fig. **3B**). Temporary clipping of the ICA allowed safe dissection and successful clipping (Fig. **3C**). Fluorescein videoangiography confirmed vessel patency (Fig. **3D**). The complete occlusion of the aneurysms was confirmed on the DSA (Fig. **3E**), and the 3D CTA reconstruction confirmed the adequate position of the clip (Fig. **3F**). The patient had no postoperative deficits.

## 4 DISCUSSION

### 4.1 Overview and Clinical Context

Kissing aneurysms (KAs) are an uncommon and under-recognized subtype of intracranial aneurysms, accounting for less than 3% of all cases in most series [8, 16]. Characterized by two adjacent aneurysms from separate arterial origins with partially adherent walls, KAs may be mistaken for multilobulated single aneurysms. This misclassification can have significant implications for both diagnosis and surgical strategy [2]. Inci and Karakaya﻿ divided the KAs into 3 types according to their position with each other on the parent artery from the surgeon’s point of view during surgery: type I (proximal/distal), type II (superior/inferior), and type III (right/left) [8] This classification allows us to highlight the main surgical difficulties in each type described, which is useful for preoperative planning and when a problem arises intraoperatively. In our series, KAs represented 2.3% of all aneurysms surgically treated, consistent with previous reports [16].

The most frequent location was the PComA–AChA segment of the internal carotid artery (60.7%), in line with earlier findings that this segment is particularly predisposed to KA formation due to complex hemodynamics and anatomical variability [8, 16]. As mentioned above, KA is a rare entity with a low incidence, so this series of 32 patients is currently the largest reported in the literature to date. In this context, our clinical experience adds to the growing literature characterizing KAs as a surgically demanding but manageable pathology.

### 4.2 Diagnostic Accuracy and Statistical Interpretation

Despite advances in noninvasive imaging techniques, preoperative identification of KAs remains challenging. In 12.5% of cases, the diagnosis was confirmed intraoperatively, underscoring the anatomical complexity and interpretive limitations of CT-A and even DSA in certain cases. Our statistical analysis did not reveal a significant association between rupture status and either sex (*p* = 1.00) or aneurysm location (*p* = 1.00), but age did emerge as a potential factor, with ruptured cases presenting at a higher median age than unruptured ones (*p* = 0.0176). This finding supports previous studies indicating that age is an important determinant of aneurysm wall stability [17].

### 4.3 Technical and Diagnostic Challenges

#### 4.3.1 Radiological Difficulties

Due to the anatomical configuration of kissing aneurysms, they are often difficult to detect with conventional imaging. Bilobed aneurysms may appear as singular lesions because of the proximity and overlap of their domes and necks [8]. In our series, the diagnosis was typically established using a combination of CT angiography and digital subtraction angiography. In select cases, 3D reconstructed angiography was instrumental in clarifying aneurysm geometry [18]. Nevertheless, four patients (12.5%) were diagnosed only intraoperatively, despite preoperative imaging efforts. These cases highlight the importance of a high index of suspicion, especially when facing irregular or multilobulated aneurysm morphology (Fig. **1**).

### 4.4 Surgical Difficulties

Surgical management of KAs presents unique intraoperative challenges. Misdiagnosis may result in the selection of a suboptimal surgical approach or failure to anticipate dual aneurysm anatomy. Our findings showed a preoperative rupture rate of 34.37% (11 patients) and an intraoperative rupture rate of 25% (8 patients), comparable to other case series involving complex aneurysms [19]. Intraoperative rupture often occurred during the dissection of adherent aneurysm walls. These findings underscore the necessity of meticulous preoperative planning and the use of tailored surgical strategies.

The selection of the appropriate approach, *i.e*., pterional, minipterional, or MAIA, was guided by lesion location and neurological condition. Intraoperative techniques, such as pilot clip application, temporary vascular control, and intraoperative fluorescein videoangiography, were essential in preventing complications and ensuring the preservation of critical vessels (Figs. **2** and **3**).

### 4.5 Clinical Outcomes and Literature Context

Favorable outcomes (mRS 0–2) were achieved in 87.5% of patients, confirming the efficacy of microsurgical treatment when conducted with experienced hands. Our series supports earlier reports that emphasize the safety and durability of open surgical clipping for anatomically complex aneurysms [16, 20]. The duration of the surgery was recorded and compared, and it was similar in all cases and not related to the functional outcome. The observed rate of poor outcomes (12.5%) was associated with high-grade SAH, vasospasm, or technical complications, scenarios that are often unavoidable despite best practices.

As Table **1** shows, while technical complexity was high, appropriate diagnosis, operative strategy, and intraoperative monitoring facilitated high success rates [8, 17, 18].

## 5 STUDY LIMITATIONS

Although this case series is the largest reported in the literature, multicenter studies or meta-analyses evaluating and comparing both the morphological characteristics of KA and its different degrees of surgical complexity are ideally needed to establish solid guidelines for the management of this rare condition.

## CONCLUSION

Kissing aneurysms are a rare and diagnostically challenging subset of intracranial aneurysms that demand a high level of suspicion and anatomical precision. Despite advances in imaging, misclassification as single bilobulated aneurysms remains common, which may delay appropriate treatment. Our series demonstrates that accurate imaging, individualized surgical planning, and intraoperative vessel assessment are essential to optimize the outcomes.

While no statistically significant associations were observed between sex or aneurysm location and rupture risk, increased age was linked to aneurysm instability. These findings emphasize the importance of individualized risk assessment and operative planning.

Greater awareness of the anatomical and radiological subtleties of kissing aneurysms may reduce misdiagnosis and contribute to safer, more effective management in specialized neurovascular centers.

## Figures and Tables

**Table 1:** Clinical, radiological, and surgical characteristics of 28 patients with kissing aneurysms.

**Characteristic**	**n or %**
Total patients	32
Sex (female)	27 (84.37%)
Sex (male)	5 (15.62%)
Mean age (years)	55.87
Age range (years)	21–73
Higher rupture risk with age (*p* = 0.0176)	Significant (Mann–Whitney U test)
Ruptured at admission	11 (34.37%)
Unruptured at admission	21 (65.62%)
Most common location: PComA–AChA	19 (59.37%)
Other locations (distal ACA, AComA, paraclinoid variants)	13 (40.62%)
Correct preoperative diagnosis	28 (87.5%)
Diagnosis confirmed intraoperatively	4 (12.5%)
Surgical approach: minipterional	14 (43.75%)
Surgical approach: pterional	15 (46.87%)
Surgical approach: MAIA	2 (6.25%)
Surgical approach: Dandy-type craniotomy	1 (3.12%)
Intraoperative rupture	8 (25%)
No intraoperative rupture	24 (75%)
Vascular anomaly present	2 (6.25%)
No vascular anomaly	28 (87.5%)
mRS 0–2 (favorable outcome)	28 (87.5%)
mRS 3–6 (poor outcome)	4 (12.5%)

## Data Availability

The data and supportive information are available within the article.

## LIST OF ABBREVIATIONS

ACA: Anterior cerebral artery
AChA: Anterior choroidal artery
AComA: Anterior communicating artery
CT: Computed tomography
CT-A: Computed tomography angiography
DSA: Digital subtraction angiography
F-VAG: Fluorescein videoangiography
H&H: Hunt and Hess scale
ICA: Internal carotid artery
KA: Kissing aneurysm
MAIA: Mini-anterior interhemispheric approach
mRS: Modified Rankin scale
MCA: Middle cerebral artery
PComA: Posterior communicating artery
SAH: Subarachnoid hemorrhage
STA: Superficial temporal artery

## STANDARDS OF REPORTING

STROBE guidelines were followed.
